# Mapping to Support Fine Scale Epidemiological Cholera Investigations: A Case Study of Spatial Video in Haiti

**DOI:** 10.3390/ijerph13020187

**Published:** 2016-02-03

**Authors:** Andrew Curtis, Jason K. Blackburn, Sarah L. Smiley, Minmin Yen, Andrew Camilli, Meer Taifur Alam, Afsar Ali, J. Glenn Morris

**Affiliations:** 1 GIS, Health & Hazards Lab, Department of Geography, Kent State University, Kent, OH 44242, USA; 2 Spatial Epidemiology and Ecology Research Laboratory, Department of Geography, University of Florida, Gainesville, FL 32611, USA; jkblackburn@ufl.edu; 3 Emerging Pathogens Institute, University of Florida, Gainesville, FL 32611, USA; mtalam@epi.ufl.edu (M.T.A.); afsarali@epi.ufl.edu (A.A.); jgmorris@ufl.edu (J.G.M.); 4 GIS, Health & Hazards Lab, Department of Geography, Kent State University at Salem, Salem, OH 44460, USA; ssmiley8@kent.edu; 5 Department of Molecular Biology and Microbiology, Howard Hughes Medical Institute, Tufts University School of Medicine, Boston, MA 02111, USA; minmin.yen@tufts.edu (M.Y.); andrew.camilli@tufts.edu (A.C.)

**Keywords:** spatial video, geographic information systems, cholera, Haiti, bacteriophage

## Abstract

The cartographic challenge in many developing world environments suffering a high disease burden is a lack of granular environmental covariates suitable for modeling disease outcomes. As a result, epidemiological questions, such as how disease diffuses at intra urban scales are extremely difficult to answer. This paper presents a novel geospatial methodology, spatial video, which can be used to collect and map environmental covariates, while also supporting field epidemiology. An example of epidemic cholera in a coastal town of Haiti is used to illustrate the potential of this new method. Water risks from a 2012 spatial video collection are used to guide a 2014 survey, which concurrently included the collection of water samples, two of which resulted in positive lab results “of interest” (bacteriophage specific for clinical cholera strains) to the current cholera situation. By overlaying sample sites on 2012 water risk maps, a further fifteen proposed water sample locations are suggested. These resulted in a third spatial video survey and an additional “of interest” positive water sample. A potential spatial connection between the “of interest” water samples is suggested. The paper concludes with how spatial video can be an integral part of future fine-scale epidemiological investigations for different pathogens.

## 1. Introduction

The water-disease nexus can be categorized in different ways [1], with arguably water-borne (diarrheal diseases and cholera) and water-related (mosquito vectored diseases, such as malaria and dengue) posing the greatest public health threat. In challenging environments, such as informal settlements or in many developing world cities, epidemiological investigations can be hindered by a lack of data. This is especially true at finer spatial scales. As a result, there are limits on our understanding of how a disease spreads, the efficacy of intervention strategies, and our epidemiological understanding of strain type and ecological presence.

Broadly, spatial investigations into this nexus can be subdivided by the pathogen, human activities in and around water, the built environment infrastructure that provides access and exposure, and physical systems that vary the amount of water flow and affect water quality. However, further complexity arises if a pathogen exists in local reservoirs, and/or human-water-human transmission.

Due to a lack of granular “explanatory” data (environmental covariates), spatial research into a disease like cholera is often performed at coarse geographic scales, with case data and environmental covariates aggregated to regions or political units, or for an ecological area, such as an estuary. For example, identifying a spatial *R*_0_, which measures the likelihood of an additional infection after a single case introduction can be important in terms of identifying variations in strain, and disease risk, which in turn should inform intervention strategies. For cholera, this has been calculated at the department scale in Haiti, with the *R*_0_ varying from 1.06 to 2.63 across spatial units [2]. However, it is plausible the spatial *R*_0_ will vary at finer spatial scales, such as by inter- or intra-settlements. Curtis and others provided a framework for a localized spatial *R*_0_ for yellow fever in New Orleans, linking household-level cases by date of infection/death and limiting transmission between cases to plausible mosquito flight ranges and the timing of cases based on the extrinsic incubation period of the virus in mosquitoes [3]. The challenge in making such a calculation for cholera is the availability of fine scale surveillance data and a means to link cases together geographically.

Even where surveillance data does exist at a fine granularity, and with the usual caveats regarding challenging environmental data quality and case undercounting, other spatial layers may be unavailable for analysis. For example, Blackburn and others considered spatio-temporal trends in 1509 household-level cholera cases reported during the 2011 epidemic in Haiti, and inferred that clusters followed inland waterways and roadways, suggesting a transportation link [4]. However, the study reached a geographic impasse, as few other data layers were available to explain connectivity between households or city blocks. As cholera transmission is amplified by human-to-human transmission, such data should include social activity, social/spatial networks, presence of hazards (water flow) and built environment infrastructure. Without detailed built or social environmental layers, it is difficult to predict intra-urban disease spread and develop appropriate intervention strategies, or ask other pertinent epidemiological questions on *Vibrio cholera* (the causative bacterium of cholera) persistence and transmission, such as how many strains are circulating within and between towns, and whether there is a change in the asymptomatic to symptomatic ratio, which have important implications with regards vaccination efficacy [5]. Answering such questions requires laboratory testing of environmental samples to determine pathogen presence (repeated sampling for persistence) and a geographic methodology to frame hypotheses and drive sample site selection.

One solution is an approach that can be used to map fine scale features associated with cholera or other diarrheal diseases. This technique also needs to be flexible enough to provide longitudinal support for iterative field work. For the investigation of diarrheal diseases, such a technique will need to be able to map localized water flow, and be dynamic enough to monitor changes. This paper will illustrate how spatial video can be used to collect and map granular water presence at the intra-urban scale, and how the resulting cartography showing risks can support on-the-ground cholera investigations using a three-stage process. Following from a 2012 surveillance of a coastal “Town A”, known to be experiencing a cholera outbreak (Stage 1), we will detail the method of a second spatial video collection in 2014, which used the maps, routes and spatial analysis of Stage 1 as a guide (Stage 2). The main difference between these two collection periods is that the second also included the collection of water samples. The results of these “prospect” samples, after lab analysis in the local field research station, are split into “of interest” and “not of interest” and then overlaid onto the water risk surfaces generated for Stage 1. These locations, after being visually validated through the spatial video of Stage 2, are used to direct a third spatial video and water sampling collection (Stage 3), approximately two months after Stage 2.

### Cholera in Haiti

Cholera continues to be an ever-present global risk, with an estimated 1.4 to 4.3 million annual cases, and 28,000 to 142,000 deaths [6]. There have been at least seven, and possibly eight, cholera pandemics since 1817. The historic trends within the pandemics are that a single clonal strain for each pandemic diffuses in geographic waves [7,8]. Within these waves are subtle changes, with variants of the same *V. cholerae* strain appearing in different continents even though still being within the same pandemic [9,10]. In other examples, similarity in genetic structure between different geographic locations might indicate either parallel evolution or an undiscovered transmission route [11]. Irrespective, the why and how of these “drifts” occur remain a point of debate [12,13].

The first cases in Haiti in 100 years began in October of 2010, along the Artibonite River [14]. As of 15 August 2015, Haiti has reported a total of 746,469 cholera cases, with 427,841 hospitalizations and 8985 deaths [15]. After passage of the initial epidemic waves, the disease has moved into a chronic, endemic pattern, with periodic/seasonal outbreaks. Unfortunately, after an initial decrease, disease burden is again trending upward, with 10,328 cholera cases, 8124 hospitalizations, and 106 deaths reported in the first three months of 2015 alone [15].

Although there has been a concerted effort by the Pan American Health Organization (PAHO), World Health Organization (WHO) and the Haitian Ministry of Public Health and Populations (MSPP) to eradicate the disease, local logistics have proven extremely challenging. Information regarding disease cases and transmission, as well as other associated factors, such as elements of the WASH cycle (water, sanitation, and hygiene) are hard to acquire. These geographic layers are not only important in understanding how and where cases occur (while also providing insight into how best to utilize intervention strategies), but are vital from an epidemiological perspective. *V. cholerae* is free-living in aquatic environments, and in endemic countries environmental reservoirs play a key role in maintaining the microorganism between annual or bi-annual human outbreaks. Changing environmental factors appear to trigger blooms of *V. cholerae* strains in these environmental reservoirs, with subsequent spill-over into human populations. However, once illness starts spreading within human populations, transmission is more direct, with the microorganism passing from one person to another within households or between households and the broader community environment.

This raises the question of the route by which such direct transmission occurs. For example, in what ways might the water-filled open drainage ditches found in many Haitian towns play a role in transmission? Might these water sources form urban reservoirs for *V. cholerae*, and/or do they provide an environmental reservoir for bacteria as they are transmitted from one person to the next? When transmission of the disease occurs between city blocks or sections of a city, information regarding social spaces (where people gather, where they eat and drink, schools, *etc.*), and infrastructure (drainage systems, water access points, *etc.*) are needed to understand the factors associated with disease spread and may serve to identify areas where decontamination efforts may prove most efficacious.

## 2. Experimental Section

### 2.1. Prior Methods and Results (Stage 1)

In 2012 an initial mapping of water risks occurred along coastal environments and towns to the west of Port au Prince, Haiti. The initial focus of these spatial videos was to map out WASH features, such as water access points, standing water, drainage trenches and pipes, human-water interaction, along with other risks, such as trash and social activity [16]. A main goal of the survey was to produce stand-alone risk maps for Haitian intra urban environments [16]. These mapped risks include standing water, trash, animals, social gathering points and key buildings, water access points, and “risky” behavior, such as children playing in drains [16].

In the previous paper [16], risk layers were created using kernel density estimation (KDE) to map the citywide distribution of standing water, trash, animals and human activity. In addition, by modifying a spatial filter approach commonly used in epidemiology, data from the spatial video were also used to create a fine scale grid, whereby the rate-of-risk was determined around critical infrastructure, such as wells and schools [16].

### 2.2. Stage 2

In 2014 a spatial video team, consisting of Kent State University (KSU) and University of Florida (UF) personnel, returned to many of the same environments captured in 2012, including Town A. Data collection occurred at approximately the same time of year so that comparisons of fine scale environmental change could be better made between the two time periods. In addition, water samples were collected in urban and estuarine environments. This was the first time water samples had been taken by the UF team from urban drainage trenches. Four Contour Plus 2 high definition video cameras were mounted on the side windows of a vehicle using suction mounts. As described by Curtis and colleagues, this high definition video has been used by the authors in multiple international environments and has proven an excellent tool to capture considerable spatial detail in otherwise data poor and challenging environments [16]. The placement of two cameras on either side of the vehicle, one pointing horizontally, the other angled down, results in a wide view shed for later digitizing. The Gobal Positioning System (GPS) path from the 2012 spatial video was used to retrace the 2012 routes, as well as add new environments of interest. Data was collected over three days in August 2014. At the end of each day all video were downloaded and daily metadata sheets constructed. This allowed for any gaps in coverage to be identified before the next day’s ride. The metadata sheet also included any notes about the day, the name of each camera, picture and GPS performance, and then a copy of the map.

Thirteen water samples were collected by the spatial video team. These locations were chosen based on the existing strategies of the UF field station, and for the first time, water filled drainage trenches, based on real-time observation of the environment *in situ*. Prior to this stage, limited resources necessitated a strategic focus on known drinking water sources such as rivers and wells. In these three Haitian sites, open drains generally offer the only separation between homes and roads. Thus, people are regularly in close proximity to these drains. They must cross over them to enter or exit homes, they walk along them due to an absence of sidewalks, and they sit near them while socializing with neighbors. In addition, research team members had observed the collection of standing water from similar sources—in Haiti and in other developing countries—for domestic uses, such as washing floors or pots and pans. Thus, while these open drains do not usually provide a source of drinking water, urban residents are in frequent indirect or sometimes even direct contact with this water. The research team selected these 13 sites based on the presence of people proximate to the source, a location that would likely see high foot traffic and the actual condition of the water source including discoloration or the presence of trash.

A brief description, photograph, and approximate time into the data collection process were recorded for each sample collection point to help verify the correct location on the spatial video path. Each water sample was then tested for presence of *V. cholerae* and cholera-specific bacteriophage. Methods used by our microbiology group for isolation and identification of O1 and non-O1 *V. cholerae* have been previously described [17,18]. In addition, samples were screened for the presence of bacteriophage lytic for clinical strains of toxigenic *V. cholerae* O1, using methods as originally described by Seed *et al*. [19]. While none of the samples tested were positive for “epidemic” *V. cholerae* O1, bacteriophage specific for clinical cholera strains were identified; the presence of such phage is generally indicative of the recent presence of clinical strains of the microorganism. For the purpose of this paper, the identification of these specific bacteriophage resulted in the sites being described as “of interest.”

**Figure 1:**
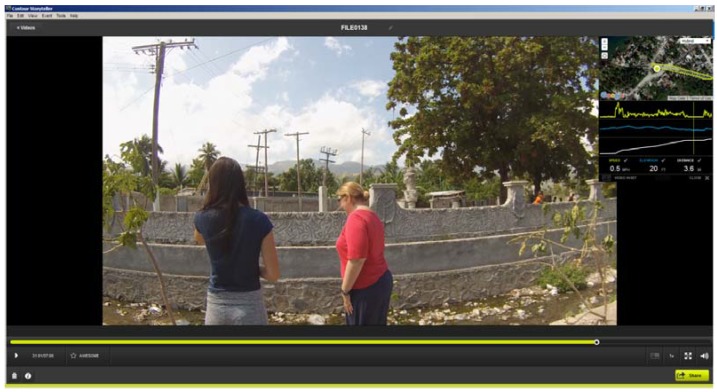
An example of Contour Storyteller software that displays both the video and its exact location (inset window). The image shows the location of a water sample collection site; notice the water and trash filled drainage trench.

As an example, Figure 1 displays two of the team at a water collection point from Stage 2. In this snapshot taken from Contour Storyteller the upper right map displays the GPS path overlaid on Google imagery. As the vehicle was stationary for approximately five minutes at each sample site, the GPS location “bounces” and typically creates a bird’s nest shape around the actual test site. The spatial average of this fluctuation gives a precise placement of where the water sample was taken. In the video image the water and trash filled testing site is clearly visible.

### 2.3. Stage 3

**Figure 2:**
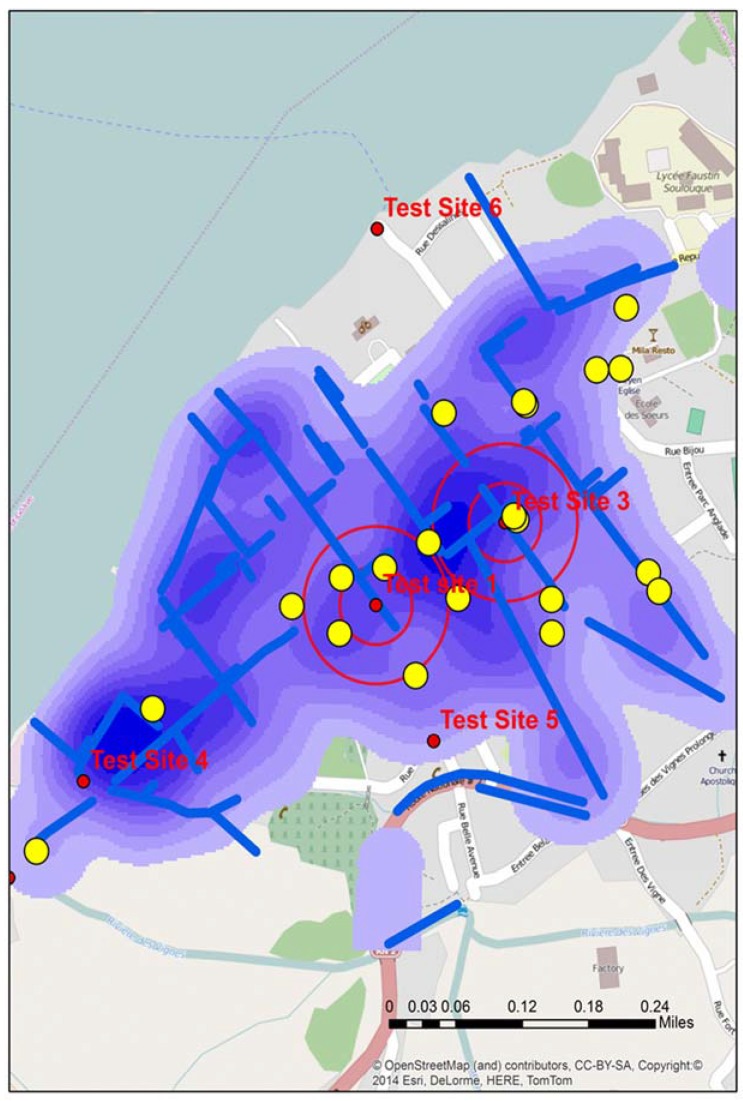
The location of all test sites in Town A during stage 2, including the two “of interest” positive sites identified by double buffers. The map also displays water risks and drainage trenches (blue), along with the locations of schools (yellow).

**Figure 3:**
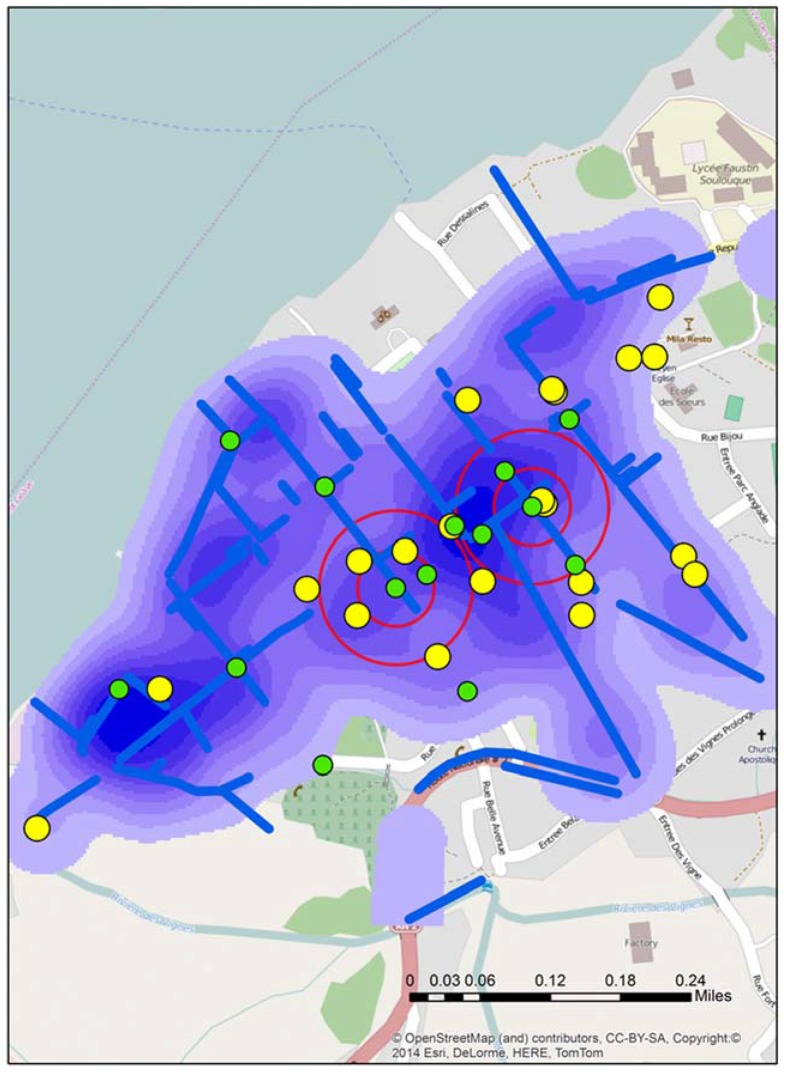
The locations of all fifteen proposed sample sites (green) for stage 3 overlaid on the local water risks (blue) and schools (yellow).

After testing of the water samples in Stage 2, analysis of those sample sites identified as being “of interest” were used to develop a sampling strategy for Stage 3. The approximate times into video recording and the brief written description were used to find the location on the associated video segment by using the metadata sheets. The location was then further tightened using the previously described GPS birds nest. Figure 2 displays these locations which were overlaid onto the water risk maps created from the 2012 spatial video run [16]. The decision making process for the new fifteen locations as seen in Figure 3 included a combination of the following; a kernel density analysis of water risk based on standing water and drains from the 2012 data, key locations such as schools or stores/vendors selling food identified in the 2012 data, and a spatial filter output grid that could be used to identify the risk at different distance bands around any location in Town A again based on 2012 data. This grid was created by using all the water risks (reduce to a point layer) as a numerator, with all digitized buildings as the denominator. The overlapping circles (filters) produce smooth rate surfaces, that can also be used to determine statistically significant intensities of risk, but, in this instance, were useful to show neighborhood risks around (for example) a school. Using these tools, with further reference to the spatial video in the 2014 collection to validate whether there was still standing water at the location, a three tier strategy was used to identify the next round of fifteen new water sample locations to be collected on a third spatial video trip:

1. Five “similar” locations were identified based on general proximity to the “of interest” sites, and the risk maps created from the 2012 spatial video run. A typical example would be a drain that had contained standing water and was “round the corner” from any positive water sample location.
2. Five sample sites were suggested by other risk locations from the 2012 hazard maps, not proximate but with some spatial connection to the “of interest” sites. For example, by looking at the elevation of the streets, it was possible to guestimate which locations might be connected to the positive sites, being either “upstream” or “downstream” in terms of water drainage. Sites were also selected based on key locations that, from previous experience, might have contributed to the positive result, such as a nearby school.
3. Five “prospect” test sites were identified from the 2012 hazard maps from which there had been no samples taken. As the water sample locations had been visually determined by the spatial video team during collection with no other risk data to guide them, it was possible that other areas might also have yielded positive results but were not tested. These five prospect sites were to fill-in such geographic gaps.

Samples were again analyzed for the presence of *V. cholerae* O1 and non-O1, and for bacteriophage specific to clinical *V. cholerae* O1 strains, as described above.

## 3. Results and Discussion

### 3.1. Stage 2

In the first spatial video collection in August 2014, approximately 95% of all previous paths from 2012 were retraced. In addition, new sites important to UF were added, including a third coastal town. In Town A [16], six water samples were collected including a transition from domiciles to the ocean, a small creek, around a water/well kiosk, roadside drains, two of which would return positive results. The locations of these testing sites can be seen in Figure 2. In Town B, which had also been mapped in 2012 but not reported in our previous publication, one water sample was taken from a large but fairly dry riverbed. In Town C, five test sites were selected. These included a well, water next to the side of the road (and a woman urinating in close proximity), and a drain with constant flowing water. Finally, an estuary was tested known to play an important role in the local cholera situation as previously identified by UF.

Figure 2 displays the location of all water test locations and schools for Town A, the locations of all schools, open water drains, and a water risk layer created from the 2012 spatial video run. Two of the test locations, A and B produced tests that were “of interest”. Two proximity buffers of 0.05 and 0.1 mile radius are centered over A and B to help with visualizing proximate risks. Additionally overlaid for comparison are the drains, a kernel density analysis of all water risks, and schools; all data were extracted and manipulated from the 2012 spatial video data collection. Both A and B water samples were taken from open drainage trenches.

As outlined above, a further fifteen sample locations were then proposed (five proximate to the two positive samples, five not proximate but connected to the positive samples, and five prospect locations). The decision making process for the new fifteen locations, as seen in Figure 3, included a combination of the following; a kernel density analysis of water risk based on standing water and drains from the 2012 data, key locations such as schools or stores/vendors selling food identified in the 2012 data, and a spatial filter output grid that could be used to identify the risk at different distance bands around any location in Town A again based on 2012 data. This grid was created by using all the water risks (reduce to a point layer) as a numerator, with all digitized buildings as the denominator. The overlapping circles (filters) produce smooth rate surfaces, which can also be used to determine statistically significant intensities of risk, but in this instance were useful to show neighborhood risks around (for example) a school.

**Figure 4:**
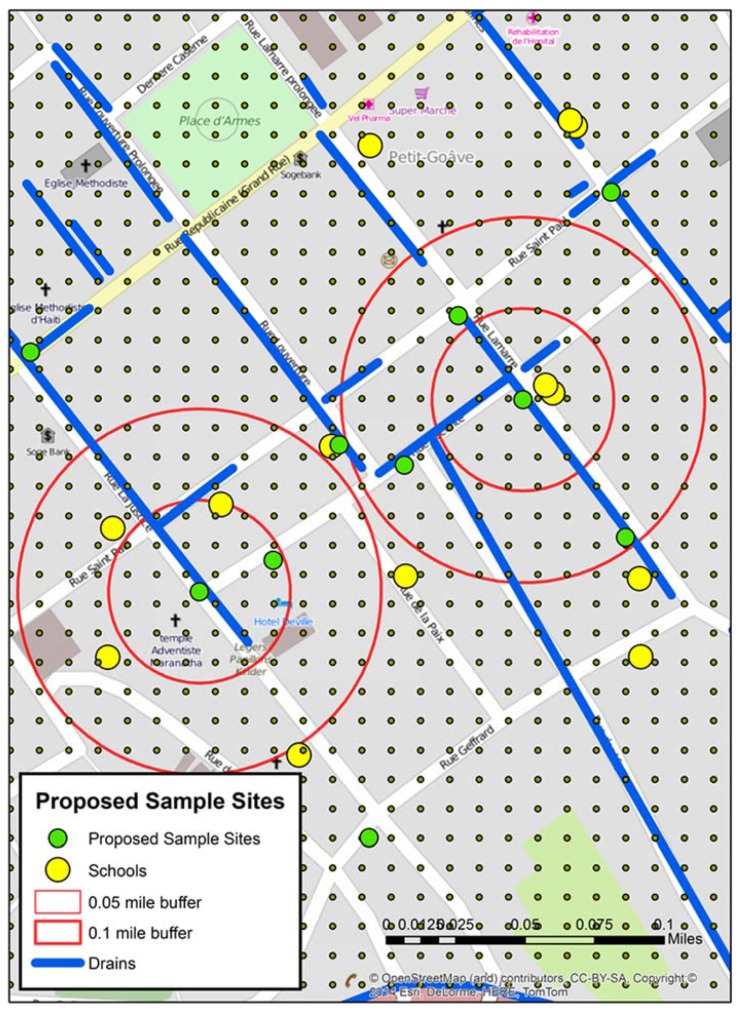
A mapped detail around the two positive “of interest” sites from stage 2 including the spatial filter grid, which was used to help determine the locations of the proposed sample sites.

Figure 4 displays the grid around A and B test sites. Within the two proximity buffers are a number of schools, drains, and seven proposed new water sample locations—some directly influenced by the previous sample sites, and others “upstream’, “downstream”, or close to possible contributing locations like schools. In addition, the spatial filter grid was overlaid. This could be queried to give the rate of water risk at any node.

For each of the proposed 15 sample sites, the 2014 spatial video was referenced to determine whether the same water risks were still present (for example the drain still contained water). If this proved to be so, then the exact latitude and longitude, a Google Earth KMZ showing the sample site and previous spatial video collection route, and a video snapshot of the location were provided to the field team at UF.

**Figure 5:**
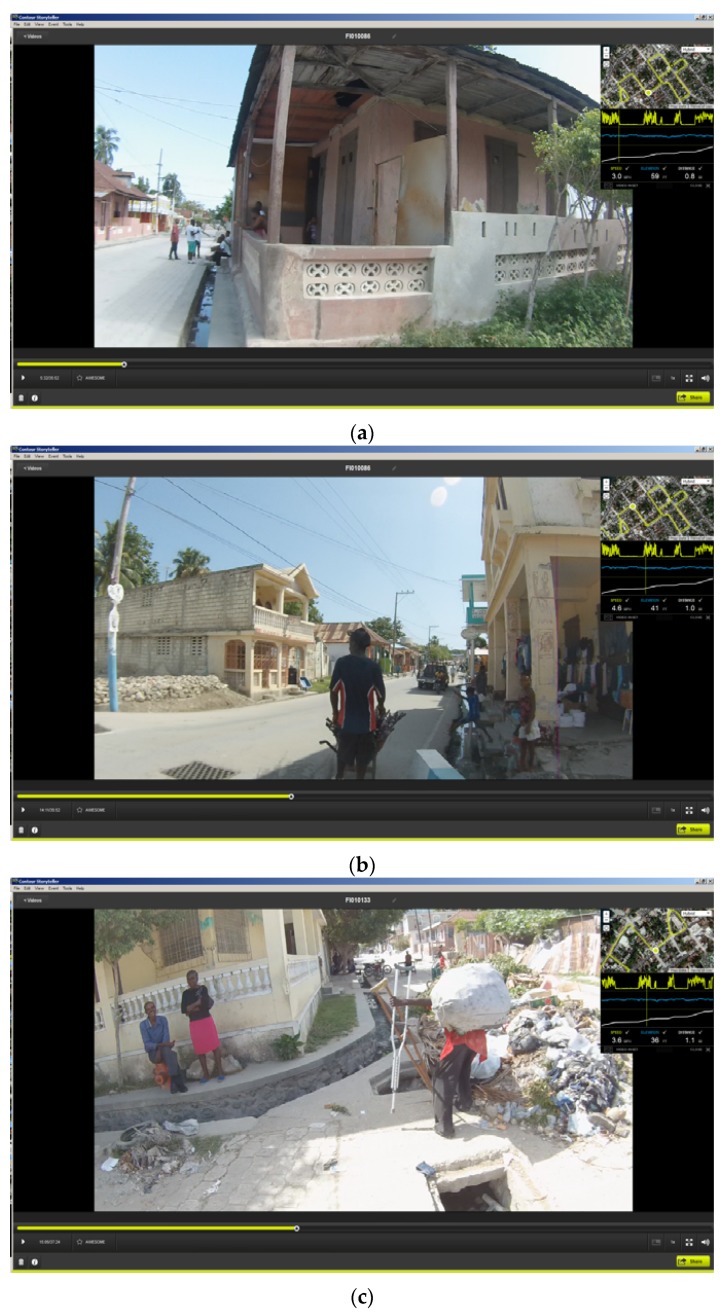
Three spatial video snapshots showing examples of where it was proposed additional water sampling should occur. (**a**): a previous positive water drain), (**b**): proximate location to a previous positive site), (**c**): a “prospect” site with obvious risk factors.

Figure 5 displays three example video snapshots supplied to the field team detailing where to collect water samples. Figure 5a shows the water drain from which a positive water sample was drawn. Notice people sitting over the drain. Figure 5b shows a suggested water sample site based on proximity to one of the positive testing sites. Notice there is a young boy leaping across the water filled drain. Figure 5c shows a “prospect” water sample location from an area of the city where no water samples were collected, but which were proximate to water risks identified in the 2012 mapping. Notice both the water filled drain and the large amount of accumulated trash.

### 3.2. Stage 3

**Figure 6:**
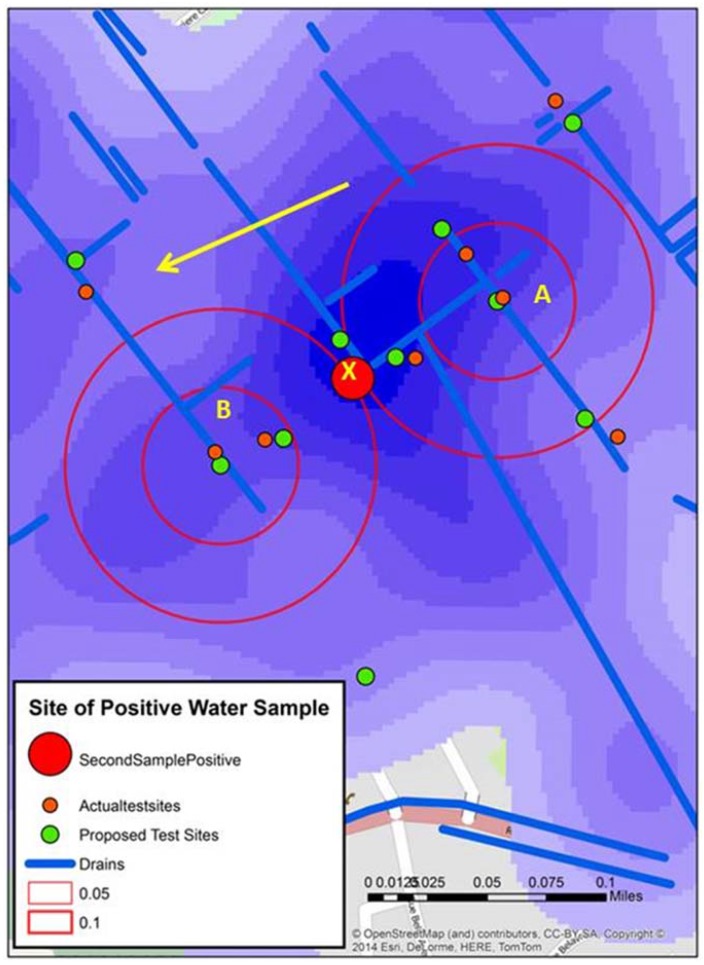
Three spatial video snapshots showing examples of where it was proposed additional water sampling should occur.

The third field trip occurred in early September 2014, with the team again retracing many of the previous spatial video routes. In addition, for Town A, fifteen water samples were collected. The locations of these around A and B can be seen in Figure 6. This map from Town A shows both the proposed test sites and where water samples were actually taken in proximity to A and B. These slight variations occurred because of several factors; a better proximate sample location being seen in the field, no need for the precision that would place proposed and actual test sites in the same place (the drain was many meters long and contained a similar amount of water), or there was a local presence, such as people being close to the proposed sample location, that resulted in a move.

Figure 6 displays the water sample from stage 3 that tested positive (X). An initial reaction is that geographically this positive site bisects A and B. However, the map reveals other possible spatial connections. X is connected to A by open drains; it is not connected to B in such an obvious manner. Continuing this line of thought, the yellow arrow shows the approximate gradient for the area; this can also be seen in one axis of the “hot spot” generated from the kernel density analysis of the water risks from the 2012 spatial video data. This might mean that water in A will flow proximate to X.

## 4. Conclusions

Prior to the new method described here, although water samples are collected locally, the pattern they reveal (and even where they are collected from) follows more spatially coarse epidemiological thinking. Traditionally those sample locations tend to be more rural or ecologically appropriate for *V. cholerae* testing locations. The rationale for such an approach is sound; if cholera transmission is driven by seasonal changes in endemic areas, as well as epidemic human-to-human transmission. However, such sampling cannot address questions aimed at understanding cholera diffusion at the intra-urban scale where pathogen persistence, water related infrastructure, water flow, social spaces (and human activity), and key locations (such as schools), all play a role. However, until now, virtually none of these spatial data layers have been available.

This paper has highlighted a method that can rectify these data deficiencies while simultaneously providing spatial field support. The spatial video provides an easy-to-use technology that can be used to generate base layers to support urban-cholera investigations, including mapping water infrastructure and human activity. The ease of collection also means that data can be collected multiple times in order to respond to environmental changes such as heavy rainfall.

By adding the simultaneous collection of water samples, robust scientific data can enrich these exploratory spatial methods. At the same time the spatial video can provide geographic and cultural context to each sample location. For example, if a sample suddenly changes the video can be re-viewed to initially gain perspective on the immediate surrounding environment, and then by comparison to other time periods, any changes can be noted. This approach can also improve field work by becoming part of an iterative process. Sample results can be tied to mapped risks and infrastructure (and even social spaces) to suggest where the next round of samples should be taken in order to build a better understanding of diffusion pathways, and eventually, possibly identifying the presence of urban reservoirs.

The results presented in this paper were simplified to highlight the methodological process—through a two stage iterative process a second “of interest” sample site was predicted as a result of two first round positive samples. The geographic (and water related) connection between these sites was suggested, and diffusion mechanics could be further hypothesized by adding visible human risks, social spaces, and key buildings. For example, by again considering Figure 4, although there is a school close to X, there are also schools proximate and upstream of A, which might support the suggested connection between the two samples. Was the school the common source of infection?

While the methods described here are at a proof-of-concept stage, it is easy to imagine how this iterative process would continue, with further detail developing the spatial connection between X and A. In addition, this technique and iterative process could be used to develop more real time epidemiological and intervention strategies to disease occurrence. An alternative strategy, and one requiring more logistical support, would be to develop a spatial sampling scheme for the entire town. However, apart from this being a more costly alternative, spatio-visual support would still be required in setting this scheme as there is no existing map of standing water, and not all drains contain water. Finally, while this paper has focused on one set of markers for cholera, there is no reason why this same approach can’t be used to support the spatial characterization of other pathogens. In this way spatial video could be used to help simultaneous investigations of diarrheal disease, and other water related infections such as leptospirosis, or mosquito-borne illnesses, such as malaria, dengue, chikungunya and of concern at the time or writing, Zika Virus.
